# Characterization of adeno-associated virus binding to alginate and its controlled release

**DOI:** 10.1039/d5na00713e

**Published:** 2026-01-28

**Authors:** John J. Amante, James R. Mandeville, Hannah Hargrove, Xiaohui Frank Zhang, Cathal J. Kearney

**Affiliations:** a Department of Biomedical Engineering, University of Massachusetts Amherst, Life Science Laboratories 240 Thatcher Road Amherst MA 01002 USA ckearney@umass.edu; b Department of Chemical Engineering, University of Massachusetts Amherst, Life Science Laboratories 240 Thatcher Road Amherst MA 01002 USA

## Abstract

Gene therapy is an increasingly explored research field with many viral vectors under study. Adeno-associated virus (AAV) is one of the more popular vectors, having already seen clinical success. While systemic or local injections are performed, more controlled means of delivery are being sought to localize treatment, reduce dosing and, minimize off-target effects. One commonly explored method is the use of hydrogels loaded with AAV placed at the site of interest. While investigating the use of alginate (a naturally occurring polysaccharide) we serendipitously discovered an interaction between the alginate itself and AAV. Through the use of atomic force microscopy (AFM) we were able to show that AAV binds to alginate and we quantified the force and frequency of the interaction. Furthermore, we have also shown that this interaction is serotype dependent, as it is not equal across different AAV serotypes. Finally, we showed that these differences in AAV serotype-alginate interactions correspondingly impact sustained release of the various serotypes from alginate hydrogels. This research offers novel insights into methods of controlled release of AAV.

## Abstract 2

In this paper we report on a novel interaction between adeno-associated virus and alginate. This AAV serotype-dependent interaction was measured using atomic force microscopy *via* single molecule force spectroscopy.

## Graphical abstract

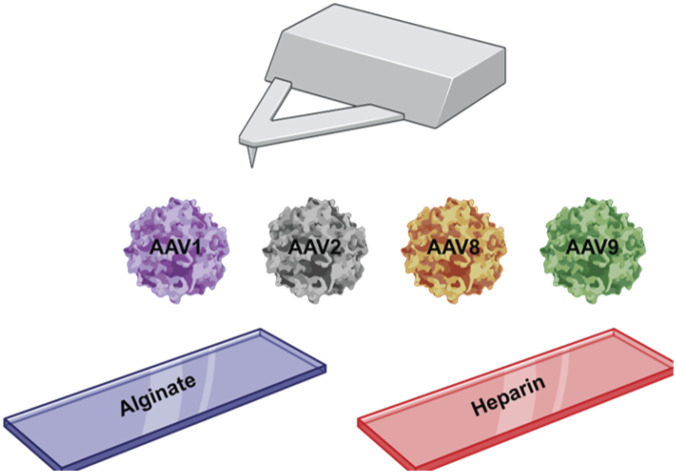

## Introduction

Gene therapy is a recently re-energized field, with increasing numbers of clinical trials and translated products emerging, and research being conducted into treating a wide range of pathologies.^1–4^ While there are a diverse number of methods of gene therapy being explored, the use of viral vectors to direct cells to regulate genes correctly is appealing, as viruses inherently have mechanisms to help them enter cells.^5^ Of the available viruses for gene therapy, adeno-associated virus (AAV) is increasingly being used, including in clinical settings.^6–9^ AAV is an attractive viral approach as it is widely considered safer than other viruses such as lentivirus or adenovirus, and it is additionally non-pathogenic.^10^ Depending on the disease, diffuse delivery of the gene therapy may be necessary (if the problem is systemic), or a more local approach may be preferable.^11–13^ In local gene therapy, controlled drug delivery systems are desirable as they can sustain the delivery of genes to the area needed over a longer period; this improves outcomes over a bolus delivery and eliminates the need for repeated dosing.^11,14^ For AAV, controlled release methods have been explored using a range of materials such as hydrogels, matrices, and stents.^15–17^ One popular hydrogel that has been tested is alginate, a natural anionic polysaccharide derived from brown algae. Alginate can be covalently or ionically crosslinked, and has found diverse uses in biomedical applications, particularly for drug delivery.^18,19^ Many groups have shown AAV can be stored in alginate and released either *via* diffusion or a trigger; however, to the best of our knowledge no one has investigated whether alginate interacts with AAV affecting its release kinetics.^17,20,21^

During preliminary investigations into AAV release from alginate for gene therapy, we observed an interplay between alginate and AAV that suggested alginate may bind AAV. To explore this interaction further, we employed an atomic force microscopy (AFM) technique called single molecule force spectroscopy (SMFS). SMFS has been a widely explored method of studying protein interactions with more uses being developed since its inception.^22–24^ To study interactions between proteins, an AFM tip is amine-functionalized with acetal–PEG–NHS (acetal–polyethylene glycol–*N*–hydroxysuccinimide), which then binds to amine groups on proteins attaching them to the AFM tip. During single molecule force spectroscopy (SMFS), the AAV coated cantilever tip is lowered down and brought into contact with the substrate on the glass surface with a constant force used for each individual trace. In such a scenario where single viruses are immobilized to the AFM tip, SMFS has sometimes been termed single-virus force spectroscopy (SVFS).^25,26^ As the tip is withdrawn from the surface, the interaction between the substrate and the biologic causes the tip to deflect. This deflection is measured through the change in the signal of the laser aligned onto the tip, and the change in the signal is translated into the force of the interaction. SMFS has previously been used to study the interaction between viruses on the tip and proteins.^33^

In this report, we wanted to determine if AAV binds to alginate and what the strength of this interaction is. Binding forces between AAV and alginate were compared to heparin, as heparin is known to bind most AAV serotypes.^27^ Initial experiments were conducted with AAV serotype 2 as it is popular for use in gene therapy applications due to its ability to transduce a wide range of cells.^28–30^ To test whether the interaction was conserved, further tests were done with serotypes 1, 8 and 9 (AAV1 and AAV8 are thought not to bind heparin^31^). We used single molecule force spectroscopy (SMFS) to investigate the binding between the virus – attached to the cantilever – and the carrier materials (alginate, heparin) were attached to glass to form the substrate. After recording the force to unbind the virus from the substrate, we used a modified Dudko–Hummer–Szabo (DHS) model to find the bond strength at zero force and identify several binding parameters.^32^ Finally, we report release of all serotypes from crosslinked alginate gels.

## Experimental

### Adeno-associated virus

AAV serotypes -1, -2, -8, -9 were purchased from Vector Builder (VB010000-9287ffw). All serotypes encoded for the same gene, enhanced green fluorescent protein (eGFP) (Vector map provided in SI Fig. 1 with permission from VectorBuilder).

### Viral transduction of cells in suspension with alginate

30k HEK293t (American Type Culture Collection (ATCC), CRL-3216) cells were plated, then 24 hours later 2.5 mg of alginate (NovaMatrix, UP LVM) was added in suspension (*i.e.*, non-crosslinked, and therefore not a hydrogel) to the cultures along with AAV2-eGFP with different multiplicity of infections (MOIs) ranging from 1 – 40. In genomic copies (gc) this is 1.26 × 10^9^–5.0 × 10^10^ gc. 2.5 mg of alginate was chosen based on typical conditions used for alginate gels in release studies (*i.e.*, 100–200 µL of 1–2% w/v alginate). After 24 h media was removed and standard culture media added to the cells. Two days post-virus addition, cells were lifted with trypsin and eGFP expression was read *via* flow cytometry in a Fortessa 3 laser flow cytometer, using a 3 laser Fortessa reading off the 488 nm laser (eGFP excitation: 488 nm) (example of gating used shown in SI Fig. 2).

### Coating of AFM tips with virus

MLCT-Bio-DC Bruker AFM tips were amino-functionalized and AAV was attached following a procedure developed by H. Gruber.^33^ 1 mg of acetal-PEG-NHS is dissolved in 0.5 mL of chloroform and 30 µL of triethylamine and cantilevers are placed in the solution for 2 hours. The cantilevers are washed with chloroform and dried with nitrogen gas. The cantilevers are then placed in 1% citric acid in water for 10 minutes, washed in water and dried with nitrogen. 2 µL of 1 M solution of sodium cyanoborohydride with 20 mM NaOH is added to 100 µL of 1–2 µM viral solution and is pipetted onto the cantilevers and incubated at room temperature for 1 hour. 5 µL of ethanolamine is added to the solution and let incubate for 10 more minutes. The cantilevers are then washed and stored in PBS. The stock virus solution used for coating was 4.64 × 10^10^ genomic copies in 100 mL of PBS. This procedure was repeated for all serotypes (AAV-1, -2, -8, -9). This protocol is summarized in SI Fig. 3.

### Coating of glass slides with biopolymers

Streptavidin (Abcam) was coated onto functionalized glass slips following the same procedure as the cantilever. Biotinylated alginate, heparin (biotinylated heparin purchased from Echelon Bioscience), biotinylated alginate was made in lab by mixing 2 grams of alginate into 200 mL of MES buffer (500 mL H20, 9.76 g MES, 8.77 g NaCl, pH set to 6.5, excess buffer stored at 4°) with sulfo-NHS (36.5 mg), EDC (18.2 mg), and biotin (100 mg). This was then deposited onto the streptavidin coated surface at a 4 nM concentration and let incubate for 15 minutes and then washed with PBS to remove unbound material.

### Atomic force microscopy experiments

A custom-built atomic force microscope was used for SMFS or SVFS.^34–36^ All measurements were conducted at 25 °C in PBS. Cantilever retraction speeds for data collection was 3.76 µm s^−1^. Force of contact between the cantilever and the glass was constant between 200 to 300 pN.^37^ Bruker MLCT-BIO-DC AFM cantilevers were used in the AFM measurements. The C tip on the MLCT cantilever was used and calibrated by measuring the inverse optical lever sensitivity through force measurements on an uncoated glass surface and the spring constant through thermal fluctuations.^38^ 60 traces were taken at one location before moving to a new location. At least 5 locations were tested for each experimental combination.

### Modelling AFM data

Igor software optimized for force pulling analysis was used to analyze force traces and to create optimal histograms. Force measurements were analyzed using a DHS model equation in an excel worksheet, to find the Δ*G* at zero force.^32^1
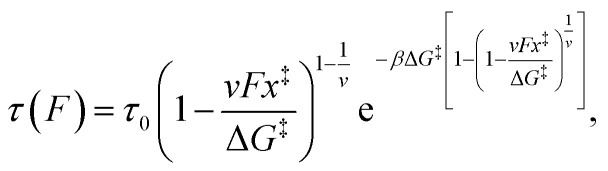


These single molecule interactions were classified as interactions that had a force of 300 pN or less.^39^ Other interactions were recorded and included in binding percentages but considered multiple interaction events. Single molecule binding events were grouped and graphed into histograms with bin widths that gave a force distribution closest to a normal distribution. By fitting these histograms on a log scale plot a linear approximation can be obtained. Fitting of the data is carried out by iteratively adjusting the three variables of consideration until the sum of the mean squared errors between *τ* measured and *τ* calculated is minimized. From this linear fitting the lifetime of the bond (τ0), calculated free energy of activation (Δ*G*^‡^), and physical distance of bond (Δ*x*^‡^) were found. Some conditions only showed multiple interactions and only binding percentage was reported from these data sets.

*τ*_0_, the intrinsic lifetime of the system, represents the length of time that the bound system would exist in an absence of outside force. So, this timescale approximates the maximum time it would take the bond between the particle and the treated cantilever tip to degrade naturally. From this value, dissociation constant of the bond (*k*_d_) can be calculated as (1/*τ*_0_). Δ*x*^‡^ represents the distance between the bound state and the transition state in meters. In other words, this variable represents the difference between the height of initial binding and the height at which rupture occurred. Δ*G*^‡^ is the apparent free energy of activation of the system in the absence of an external force. This is the amount of energy in *N* × *m* that would be required to break the bond if no force were to be applied. Energy in that context could be any non-force application, such as an external heat source.

### AAV-eGFP release from crosslinked alginate gels

To investigate if these differences in forces and binding efficiencies had consequential effects for release of viruses from alginate hydrogels, alginate gels (1% w/v) were loaded with 1 × 10^9^ gc of either AAV1, AAV2, AAV8, or AAV9 all encoding for eGFP. Gels were cast as previously described.^40^ Briefly alginate was mixed with AAV and stored in the syringe for 60 min. Next, alginate was crosslinked with calcium sulfate and injected into a collagen-GAG scaffold to simulate its use in a drug delivery-tissue engineering model.^41^ After 30 minutes, wells were flooded with media and given 24 hours to diffuse then media was collected and fresh media added; this was repeated every 24 h. Conditioned media from days 1, 3, and 5 was added to HEK293 t cells (30k cells). Transduction efficiency was used as a measure of release and was quantified *via* flow cytometry as described above.

### Motion-enhanced release

To see if the low release for AAV8 could be overcome by enhancing diffusion *via* ultrasound, we repeated release experiments with AAV8-eGFP. On day 1 the whole scaffolds were placed in a sonicating water bath (Emerson Branson) for 2 hours. Then the conditioned media was added to cells and transduction efficiency recorded.

### Statistics

For alginate virus interference experiments, most probable binding forces, and lifetime of bonds One-way ANOVA was run with Tukey *post hoc* analysis. For release of different serotypes 2-way ANOVA was run with Tukey *post hoc* correction. When comparing binding efficiency between substrates for each serotype and motion *versus* motionless release a Students' *t*-test was performed. Statistics were performed in Graphpad and *p* < 0.05 was considered significant.

## Results

### Alginate in suspension inhibits AAV2 transduction

**Fig. 1:**
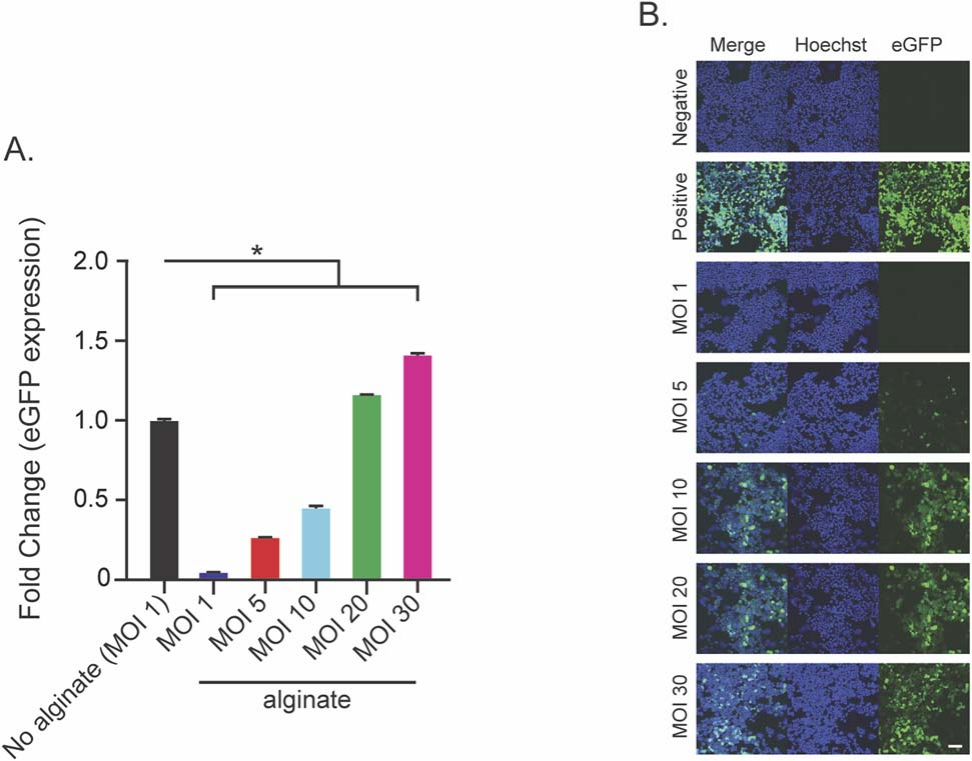
Alginate interferes with adeno-associated virus uptake. (A) 2.5 mg of alginate in solution was added to HEK293t cells with increasing amounts of AAV2-eGFP. (A) MOI of 20 was needed to reach the eGFP expression seen in the positive control. (B) 20× pictures of cells treated with various MOIs and alginate (scale bar = 100 µm, images taken 48 hours after transduction; enlarged images provided in SI Fig. 4).

At a fixed amount of alginate (2.5 mg) suspended in media over cells, we loaded AAV2-eGFP at increasing MOI = 1–40. At lower MOI (1–10), there was a significant decrease in the percent cells transduced (4.0–4.5% of positive control) (Fig. 1). However, by increasing the MOI to 20 we could achieve eGFP expression that matched the alginate-free positive control (*i.e.*, “No alginate, MOI = 1”). As alginate had a dramatic inhibitory effect on viral transduction with AAV2, this data suggested that there was some interaction (investigated further below) between alginate and AAV that could be overloaded with sufficient AAV2. Since alginate is a linear polysaccharide polymer, we hypothesized this was due to saturating the binding regions of the AAV. Heparin sulfate, which is a similar linear polysaccharide to alginate, has previously been shown to bind to AAV2.^42^ While suggestive of binding, this data did not definitively show AAV interacts with alginate, so we next moved to AFM virus–protein interaction experiments.

### Most probable binding forces

**Fig. 2:**
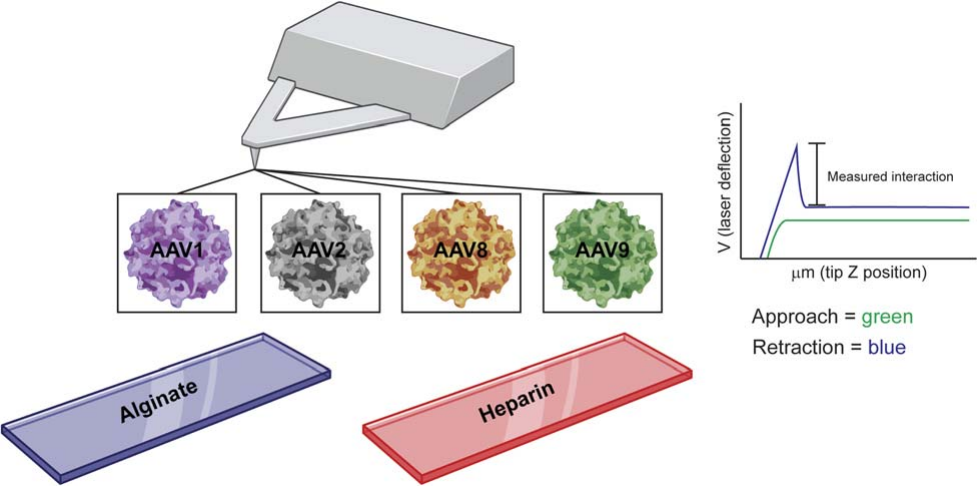
Schematic of workflow. Various serotypes of AAV (all with 4.64 × 10^10^ genomic copies) were bound to the AFM cantilever tip *via* biotinylation. This was then put in contact with glass slides coated with either alginate or heparin (again bound *via* biotinylation, at a concentration of 4 nanomolar). Then the tip was raised and the force on the cantilever was measured throughout.

**Fig. 3:**
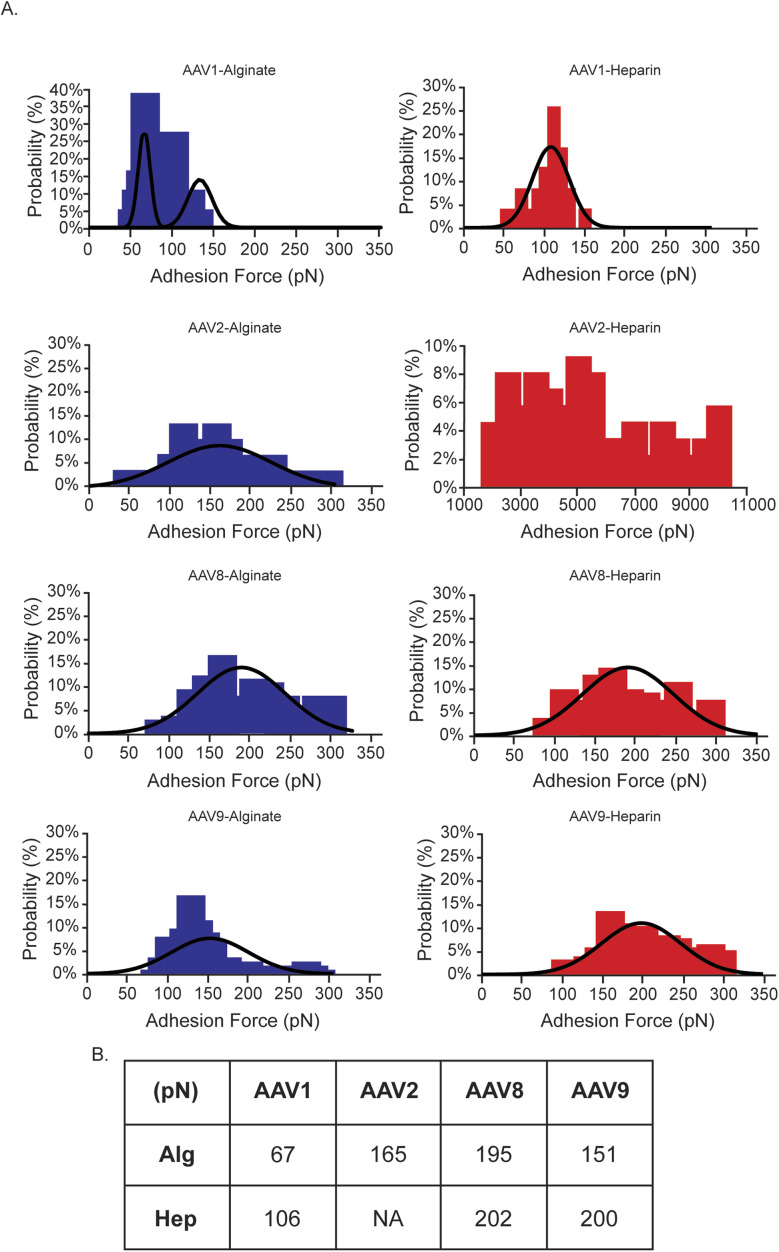
Most probable binding forces. (A) The calculated most probable forces for the interactions between heparin (left column) alginate (right column), and all serotypes. Probability curve for all possible graphs included. AAV1–ALG interaction had two distinct peaks with the first being a single interaction and the second being two interactions. (B) The most probable binding force for each condition that it could be found in.

To test the binding interaction between alginate and AAV, we used an atomic force microscopy approach called SMFS (Fig. 2). AFM cantilever tips were coated with AAV virus and brought into contact with alginate or heparin coated slides (heparin was used as a positive control). These experiments allowed us to assess single molecule interactions between the AAV and alginate. To broaden our understanding of AAV–alginate interactions, we included AAV1, AAV8, and AA9, as well as AAV2 in these experiments. For all serotypes tested, interactions between AAV and alginate or heparin substrates were observed during force pulling experiments and used to calculate the most probable binding force (Fig. 3A). The exception was interactions of heparin with AAV, where the observed adhesion force was above our 300 pN threshold set for single molecule interactions. Otherwise, single molecule interactions were observed for all combinations of AAV serotype and alginate/heparin (values are reported in Fig. 3B). The most probable binding forces ranged from 67–195 pN for alginate and 106–202 pN for heparin. AAV8 had the highest binding force for both alginate and heparin (195 pN and 202 pN respectively), being significantly higher than any other serotype (*p* < 0.05). AAV2 had the second highest binding for alginate (165 pN), followed up by AAV9 (151 pN), with AAV1 having the weakest probable binding force with alginate (67 pN). Notably, the AAV1 alginate interaction showed two distinct peaks below the 300 pN threshold with the peak of the second having a most probable force of 116 pN. While we are unable to calculate a probable binding force for AAV2-heparin, AAV1–heparin (106 pN) has a weaker binding than AAV9–heparin (200 pN). All values are statistically different from each other (*p* < 0.05).

### Binding percentage

**Fig. 4:**
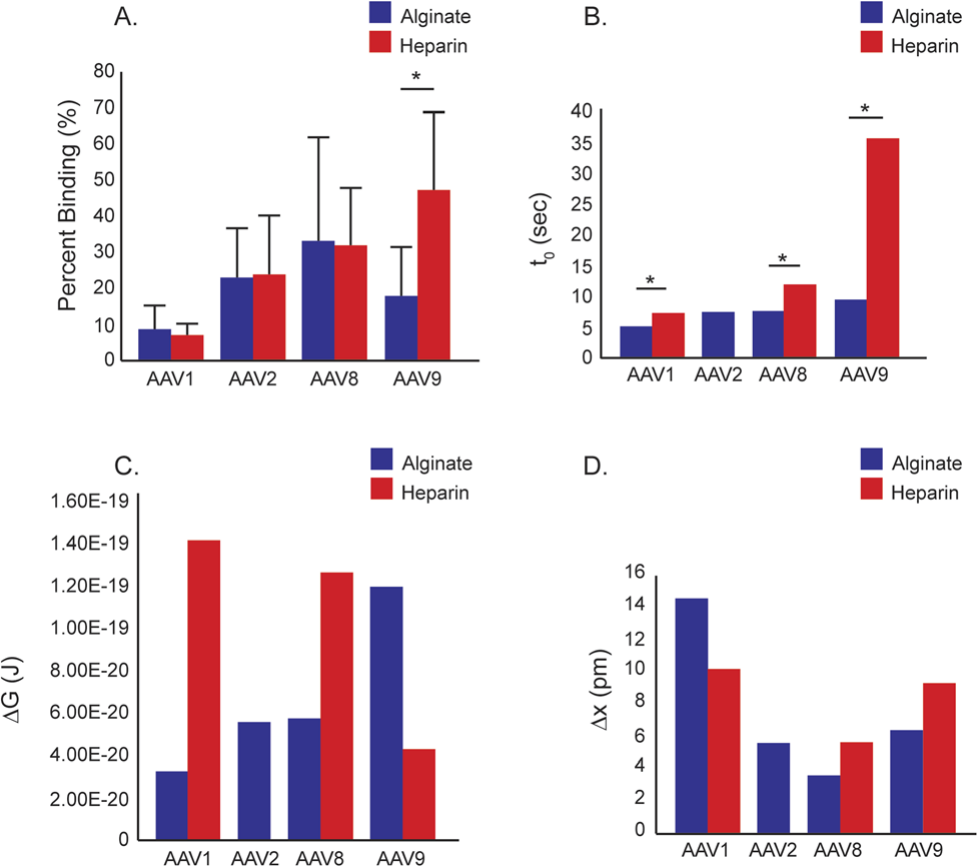
Calculated values from AFM data. (A) The percent binding for each serotype and substrate was calculated. Statistically there was only a significant difference between heparin and alginate with AAV9. (B) The lifetime of the bonds were analyzed, with all values being generally the same except AAV9–heparin. (C) The calculated free energy of each bond. (D) The distance each interaction can reach before rupture.

The binding percentage, which is a measure of what percentage of runs contained an interaction, demonstrated an affinity of each serotype for both heparin and alginate (Fig. 4A). As a negative control, we tested the binding events for AAV2 and a BSA-coated glass slide and recorded a negligible binding rate of 2.5%. While not statistically significant between the alginate groups, binding percentages varied with serotype. For heparin, we saw a similar trend across serotypes (3% or less difference between alginate and heparin), with the exception of AAV9 which had a significantly higher percent binding for the heparin group when compared with the alginate (30% difference). For alginate, binding percentages were the lowest for AAV1 (8.75%) and highest for AAV8 (32%), with AAV2 and AAV9 between these values. The serotype–substrate interaction with the highest binding, however, was AAV9–HEP with a 47.3% binding rate. AAV2 also was tested with chitosan, but ultimately chitosan was dropped from further study (SI Fig. 5).

### Lifetime of bonds

With the exception of the AAV9–heparin interaction (35.9 seconds) all lifetimes range between 5.0 and 11.9 seconds (Fig. 4B). AAV9 also has the longest *τ*_0_ with alginate (9.5 seconds) of all serotypes. AAV8–alginate and AAV2–alginate are almost identical at 7.6 seconds and 7.4 seconds respectively. AAV1–alginate has to lowest *τ*_0_ with 5.0 seconds. As stated previously AAV9–heparin was the longest *τ*_0_ of all conditions, while AAV8–heparin was 11.9 seconds and AAV1–heparin was once again the shortest *τ*_0_ per substrate at 7.3 seconds. Each serotype is statistically different between substrates (*p* < 0.05).

### Free energy of activation

Using the DHS model (eqn (1)), the calculated free energy of activation in the absence of force, Δ*G*^‡^ was calculated for conditions that exhibited single molecule interactions (Fig. 4C).^32^ AAV1, AAV2, and AAV8 showed similar Δ*G*^‡^ for the alginate interactions, between 3 × 10^−20^ J and 6 × 10^−20^ J with heparin interactions for AAV1 and AAV8 in the range of 1.3–1.4 × 10^−19^ J. AAV9, as with binding percentages, exhibited significantly different behavior, with heparin showing similar Δ*G*^‡^, 4.30 × 10^−20^ J, as the alginate interactions with other serotypes and the alginate interaction similar to that of the heparin in the other serotypes with a Δ*G*^‡^ of 1.2 × 10^−19^ J.

### Δ*x*^‡^

Interestingly AAV1–alginate had the longest physical bond (14 pm) despite having both the lowest frequency and strength of bonds (Fig. 4B). This shows that the bond is very elastic. The Δ*x*^‡^ for AAV1–heparin is the second longest of the tested interactions (10 pm), whereas the other interactions were the same scale to each other (3–9 pm). From our SMFS results we were able to get an average binding efficiency for each condition as well as calculate the lifetime of the bond and calculated free energy of activation. Overall, the binding data for alginate considered collectively suggests that AAV1 has the lowest interaction with alginate, and AAV8 has the highest. AAV2 is in between these values, as is AAV9; however, AAV9 has the highest Δ*G*^‡^ of the alginate groups. To the best of our knowledge no one has investigated the calculated free energy of activation for alginate with any material. For Heparin, AAV1 had the lowest binding force and binding percent, with AAV8 and AAV9 being similar. While the AAV–heparin interaction has been previously reported we were unable to find any papers that quantified the force of the interaction^27^

### Effect of changing ionicity and pH on interaction

**Fig. 5:**
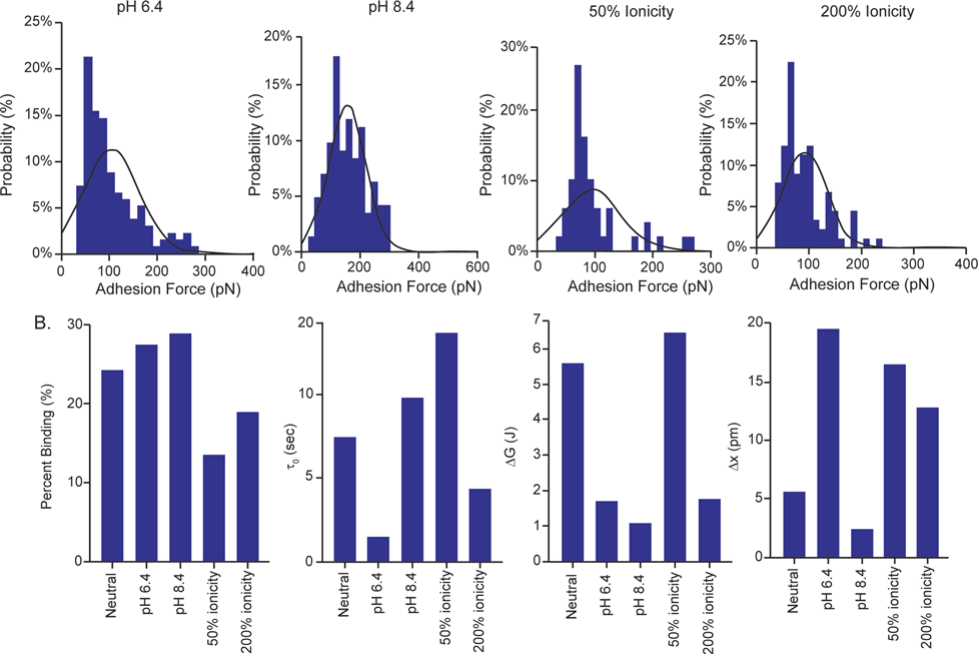
Most probable binding force and calculated values from AAV2–Alginate interactions under altered charge and pH levels. (A) The calculated most probable binding forces for altered ionicity (50% and 200%) or pH (6.4 and 8.4). (B) Calculated free energy of bonds, lifetime of bonds, percent binding, and stretching distance for these conditions, with the data from the standard experimental AAV2–Alginate groups included for reference.

To begin to understand the type of interaction observed, we conducted further experimentation between AAV2-coated tips and alginate by adjusting the ionicity (using NaCl) to 50% and 200% of the experimental values, or the pH to 6.4 or 8.4. When the ionicity of the media was changed, the binding force was equal between 50% and 200% ionicity; however, the free energy of the bonds and the lifetime of the bonds were increased at lower ionicity, with the opposite observed at higher ionicity (Fig. 5A). This is consistent with there being an electrostatic component to the interaction, as decreasing the ionic strength of the media enhances this interaction. At the higher pH, the most probable binding force, the lifetime of the bond, and the percent binding increased (however, the free energy of the bond decreased) (Fig. 5B). While we cannot directly conclude that this is attributable to conformational change, the changes in binding properties observed at both pH values suggest that the interaction is altered by changes in pH, which is known to affect conformation. Therefore, we propose that the interaction is non-covalent, and a combination of an electrostatic interaction and a conformational interaction, but acknowledge that further testing is needed to fully interpret this.

Using AFM, we measured the binding efficiency and other key metrics related to both the AAV–alginate and AAV–heparin interaction. Increasingly in literature, alginate is being used to release AAV.^17,20,43^ However, reports to date do not note the potential of AAV–alginate interaction, perhaps due to the amount of AAV used relative to the alginate concentration. For example, in two different reports from the Onoe group AAV5 and AAV1 was used, which has been shown to not bind heparin.^44^ While the Silva group used AAV2 they started with a higher concentration in their alginate hydrogels, given our data showing a sufficient amount of AAV can saturate the binding they may have started with the binding interaction already saturated before collecting any release data. The Müller group used a AAV9 variant, raising the possibility they might have removed the binding component from the AAV.^21^ Alginate had been shown to bind other viruses (*i.e.*,SARS-CoV-2) but we were unable to find any reports showing this with any AAV serotype.^45,46^ Ultimately, we wanted to test if our observed binding interaction had consequential effects on biomaterial-controlled release of AAV by characterizing different AAV serotypes release from alginate.

### Serotype of AAV affects release from alginate hydrogel

**Fig. 6:**
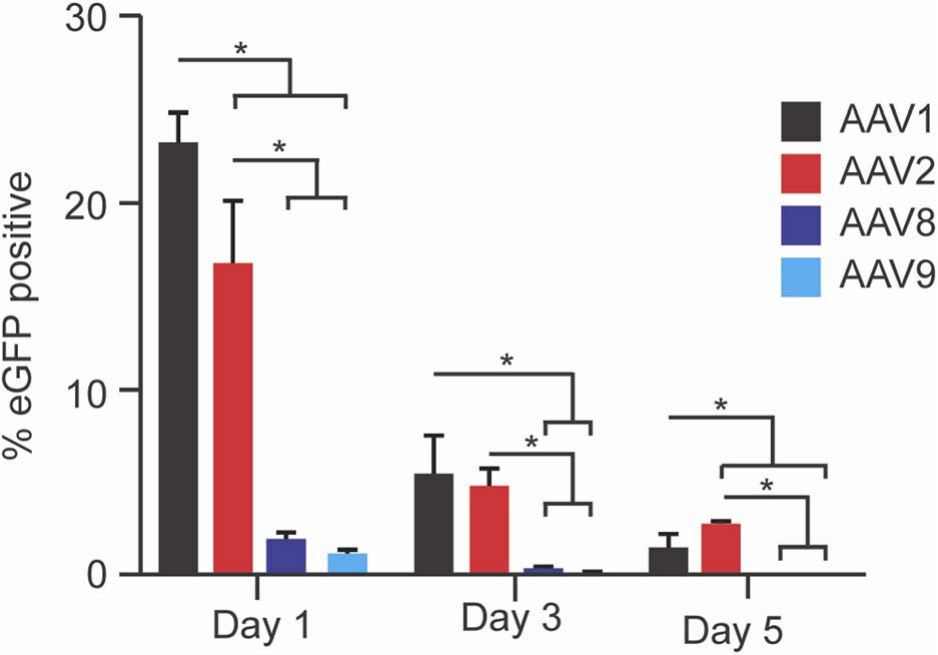
AAV–alginate binding has an observable effect on release. AAV serotypes were loaded into crosslinked alginate hydrogels in collagen–GAG scaffolds and allowed to diffuse over five days. Conditioned media was then added to HEK293t cells and fluorescence was measured *via* flow cytometry. AAV2 and AAV1 had the highest expression, while AAV8 and AAV9 had barely detectable expression.

To see if the binding data was consistent with the practical application of alginate as a drug carrier for local delivery of AAV, we conducted release experiments from crosslinked alginate gels. Bioactive AAV1 and AAV2 were released from the alginate hydrogels and successfully transduced cells over all five days (averaged transduction percentage for day 1, 3, and 5 were: AAV1 = 23.24, 5.42, 1.51%; AAV2 = 16.62, 4.89, and 2.76% respectively). Interestingly, AAV8 and AAV9 demonstrated negligible release and transduction (averaged transduction percentage for day 1, 3, and 5 were AAV8 = 1.97, 0.41, 0.13%; AAV9 = 1.24,0.23, 0.06%) (Fig. 6). This suggests a combination of binding efficiency, calculated free energy of activation, and binding force all factor into AAV release from alginate hydrogels. This has large implications for AAV delivery, as combining alginate with different AAV serotypes will give unique release profiles, and may be prohibitive in some circumstances. One-way ANOVA reveals AAV1 and AAV2 always had a statistically higher release than AAV8 and AAV9 (*p* < 0.05), but only on day 5 was there a statistical difference between AAV1 and AAV2. The observed binding effects have consequences on AAV release from ionically crosslinked alginate hydrogels, as shown here. While AAV1 and AAV2 saw high release over 5 days AAV8 and AAV9 saw minimal release. Release appears to be determined by more than binding efficiency as AAV2, AAV8, and AAV9 had similar binding efficiency yet display different release profiles. Our interpretation is that the binding force also significantly affects release as AAV8 and AAV9 had a higher binding force than AAV2. In these experiments, we have switched from alginate molecules to a crosslinked hydrogel, so it is necessary to also consider the impact of the mesh on release. Importantly, past work has shown that 1% alginate gels produce a mesh size in the range of ∼50–150 nm, which is larger than AAV (∼25 nm diameter).^20,47^ Given that these are reasonably similar to each other, the diffusion coefficient will be impacted for the virus travelling through this meshwork; this will additionally impact release kinetics beyond the binding effects described herein. However, previous data has shown the ability of AAV to diffuse from alginate.^20,40^

### Motion-enhanced release

To see if ultrasound stimulation could be used to break the bonds between AAV8 and alginate, we tested whether ultrasonication could enhance release. Despite the addition of ultrasound there was no significant difference of AAV8 release when compared to standard diffusion (SI Fig. 6), suggesting higher amounts of energy are required to break the bonds between the AAV8 and alginate. Due to all of these calculations assuming a system without motion, we hypothesized introducing some energy into the system would change the release profile. Interestingly this was not the case as ultrasonicating a hydrogel loaded with AAV8 did not increase the release as compared to a motionless system, and we expect that higher energy input is required.

Using the various metrics for binding and considering them holistically, AAV1 appears to have the lowest interaction with alginate, AAV8 the highest, with AAV2 and AAV9 lying between these; we note, however, that AAV9 has the highest Δ*G*‡. This serotype-specific effect is further supported by the release data, whereby eGFP expression is higher for AAV1 and AAV2 *versus* 8 and 9 following release from crosslinked alginate gels. This work supports the previously undescribed finding that there is a serotype-specific interaction between alginate and AAV serotypes 1, 2, 8, and 9. Heparin was included as a positive control due to its known interaction with some AAVs. Additionally, alginate is known to bind heparin binding proteins, so there is likely crossover in the interaction.^11^ As expected based on literature, AAV1 did not bind heparin with good efficiency or force.^31^ What was unexpected was observing binding between AAV8 and heparin as previous attempts to use heparin to purify AAV8 have not worked.^48^ While we cannot speak to its use as a purification agent we have shown that AAV8 does bind heparin. We were unable to find a paper that tested heparin binding to AAV9 making the observed binding a novel interaction. While we did not test different alginate sources or batches herein, the heterogeneous nature of the G and M block distribution within this naturally-derived polymer ensure a random sampling of G- and M-block interactions is included. However, testing with different combinations may help tune the effect further. This report has implications in the field of gene therapy and drug delivery. For select applications, we suggest studying the specific interaction between AAV and alginate to tune the release profile. With many naturally occurring serotypes as well as an increasing number of lab produced serotypes, there are plenty of candidates to try for the preferred profile.^49^ This work also has interesting implications for alginate's use as an antiviral agent. While we are the first to report an AAV–alginate interaction, alginate has been shown to have antiviral properties for some viruses, and suggests examining other viruses in future work.^45^ While we have not identified where on the virus alginate is binding, understanding this could enable its more specific use as an antiviral. Additionally, understanding the exact binding mechanism could prove valuable in the design of new release systems and/or the appropriate choice of carrier polymers. While single molecule force spectroscopy was employed to examine the binding effect in this work, other techniques (*e.g.*, ELISA or Isothermal Titration Calorimetry) can be employed in future work to further confirm and investigate this interaction.

## Conclusions

In this report we identified a novel interaction between AAV and alginate. Using a modified DHS model and AFM we determined several metrics related to this interactions (*τ*_0_, Δ*x*^‡^, Δ*G*^‡^) and showed that the AAV–alginate interaction is serotype specific. These binding effects had a consequential effect on serotype-specific AAV release from alginate hydrogels. This report has implications for long term control release studies involving custom AAV serotypes.

## Author contributions

JJA: conceptualization, data curation, formal analysis, investigation, methodology, project administration, validation, visualisation, writing – original, review, and editing. JRM: data curation, formal analysis, investigation, methodology, validation, visualisation, writing – original, review, and editing. HH: data curation, formal analysis, methodology, validation, writing – review and editing. XFZ: formal analysis, funding acquisition, methodology, project administrations, resources, software, supervision, writing – review and editing. CJK: conceptualization, formal analysis, funding acquisition, methodology, project administrations, resources, software, supervision, writing – review and editing.

## Conflicts of interest

There are no conflicts to declare.

## Supplementary Material

NA-OLF-D5NA00713E-s001

## Data Availability

Data for this article, including data for all figures are available at ScholarWorks@UMassAmherst at https://doi.org/10.7275/ctem-5d94 Supplementary information (SI) is available. See DOI: https://doi.org/10.1039/d5na00713e.
