# Supplementary material for: Characterization of adeno-associated virus binding to alginate and its controlled release
Source: Nanoscale Adv. 2026 Jan 28;8(5):1739–47. doi: 10.1039/d5na00713e (PMC12893933; doi:10.1039/d5na00713e)
Supplement: NA-008-D5NA00713E-s001 [file NA-008-D5NA00713E-s001.pdf]

Supplementary Information (SI) for Nanoscale Advances.
This journal is © The Royal Society of Chemistry 2026

Characterization of adeno-associated virus binding to alginate and its controlled release

John J Amante[a*], James R Mandeville[a*], Hannah Hargrove[b], Xiaohui Frank Zhang[a], and Cathal J Kearney[a]

a. Department of Biomedical Engineering, University of Massachusetts Amherst, Life Science Laboratories, 240 Thatcher Road, Amherst, MA, 01002
b. Department of Chemical Engineering, University of Massachusetts Amherst, Life Science Laboratories, 240 Thatcher Road, Amherst, MA, 01002

Supplementary Information

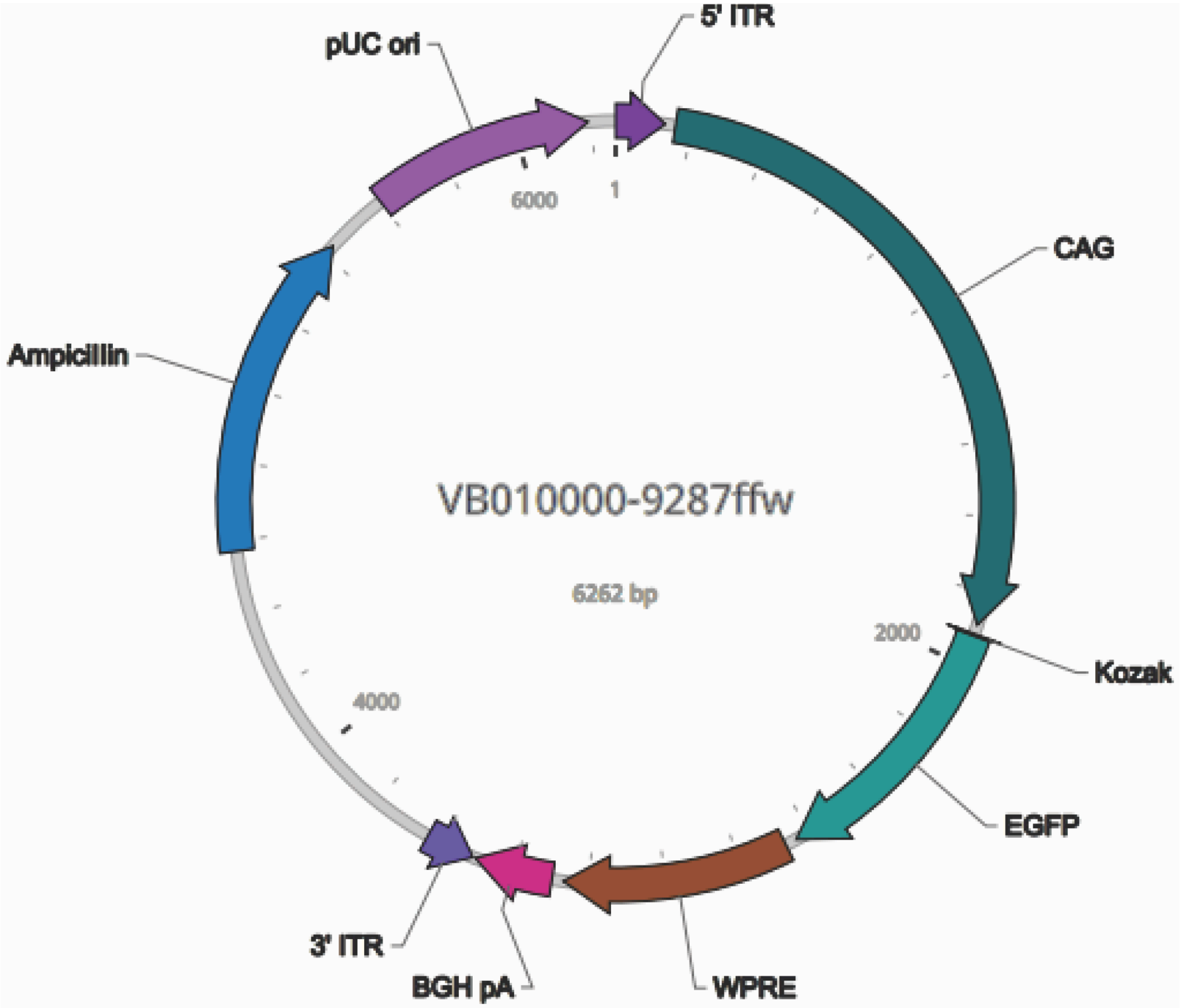

Supplemental Figure 1. Vector map of plasmid used in all AAV serotypes as provided by VectorBuilder.

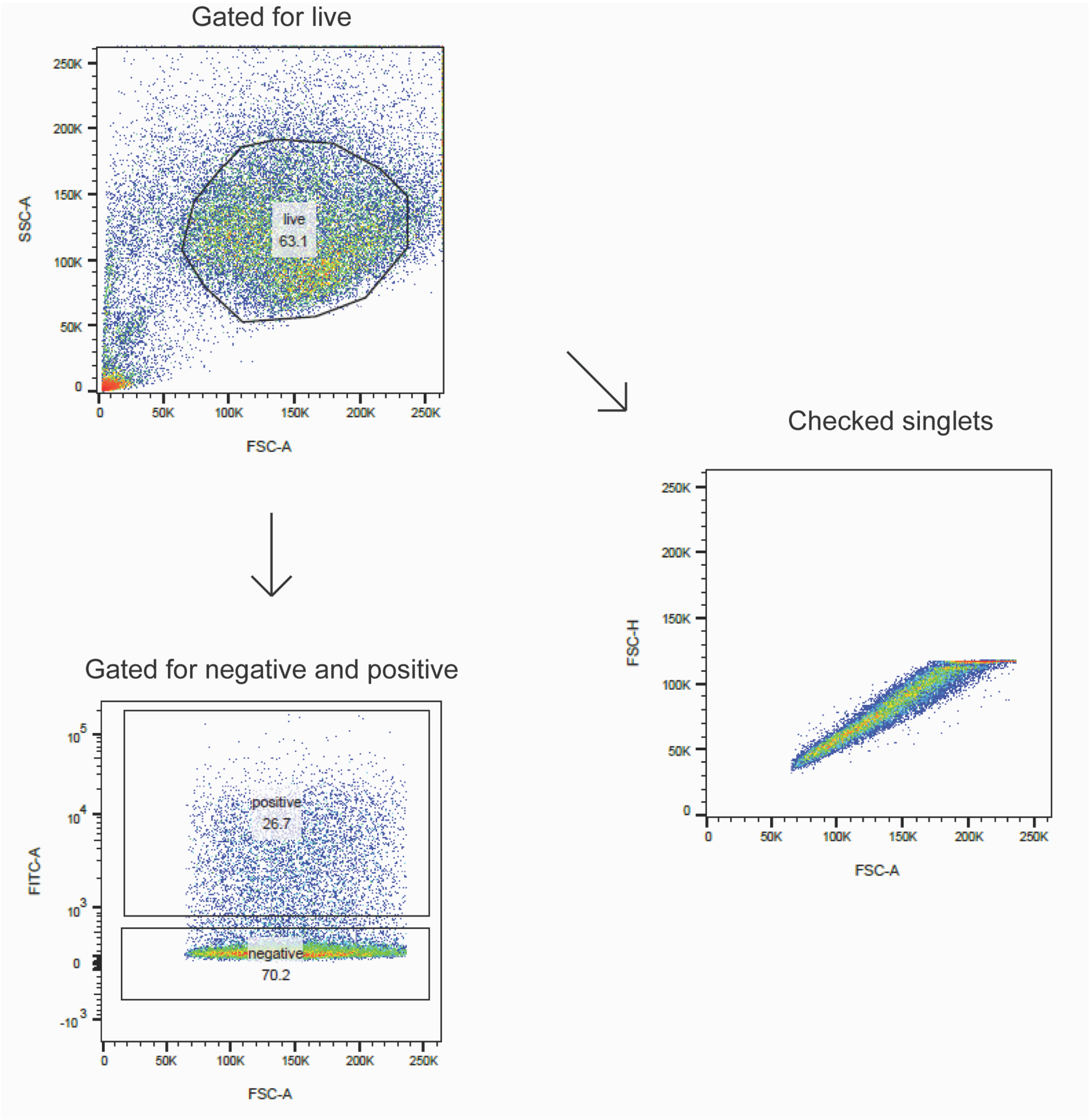

Supplemental Figure 2. Example of flow cytometry analysis. First, cells were gated on the SSC-A vs FSC-A axes for live cells. Then that population was gated for negative (gate set by using negative control) and positive (gate set by including area above negative gate). The live population gate was also used to confirm cells were in single suspension.

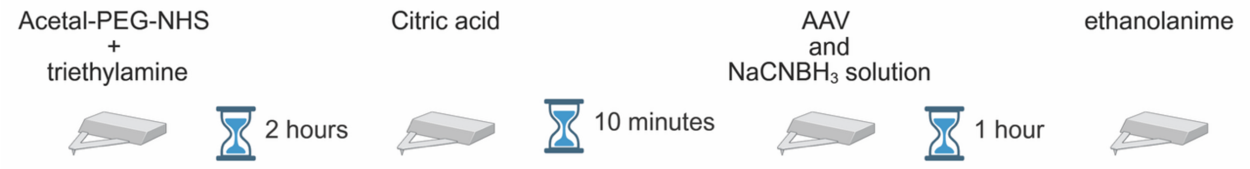

Supplemental Figure 3. Functionalization of the AFM tip. First 1 mg of Acetal-PEG-NHS is dissolved in 0.5 ml of chloroform. This is then mixed with 30 μl triethylamine and the cantilever is placed in the solution. After 2 hours, the cantilever is removed and washed with chloroform (3 times, 10 minutes each, dried with nitrogen gas). The cantilever is then immersed in 1% citric acid for 10 minutes before being washed in water (3 times, 5 minutes each, dried with nitrogen gas). The cantilever tip is then covered with AAV solution and 2 μl of $NaCNBH_3$ solution is added. After 1 hour, 5 μl of ethanolamine (1 M, pH 8.0) is added to the tip, incubated for 10 minutes, then washed 3 times in PBS. The tip is now ready to be mounted within the AFM.

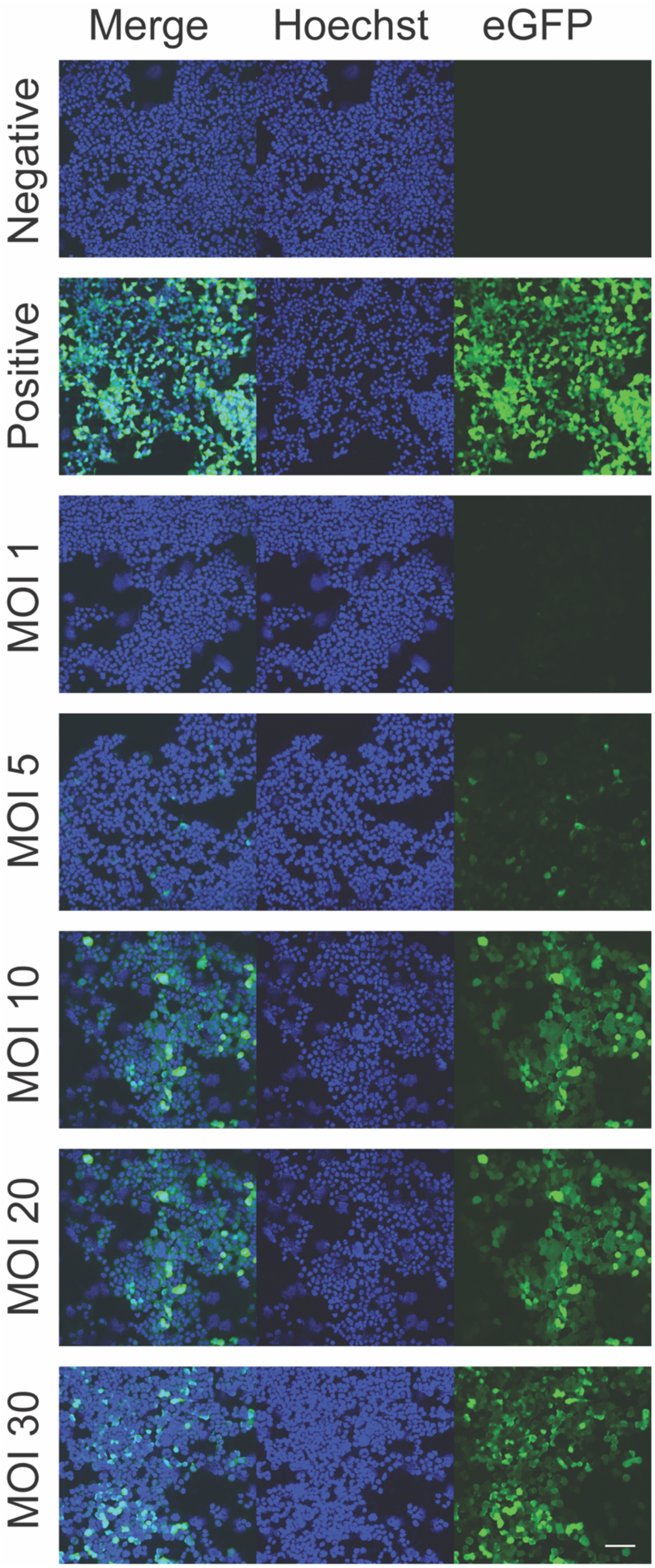

Supplemental Figure 4. Enlarged version of Figure 1B. With the exception of the positive control (no alginate, AAV2-eGFP at MOI = 1) and negative control (no alginate, no AAV2-eGFP), HEK293t cells were treated with alginate (2.5 mg) and increasing MOIs from 1 to 30. Images are taken 48 h after the addition of alginate and AAV (scale bar = 100μm).

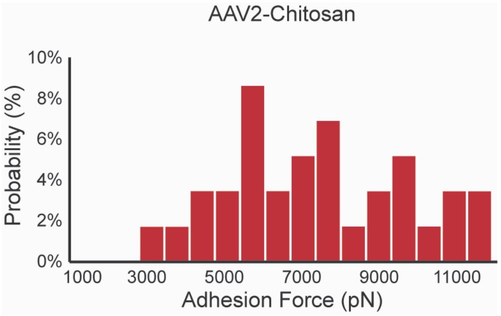

Supplemental Figure 5. Most probable binding forces between AAV2 and chitosan. Chitosan is a positively charged naturally-derived polymer. Further study into AAV binding chitosan was ultimately abandoned as binding efficiency was inconsistent.

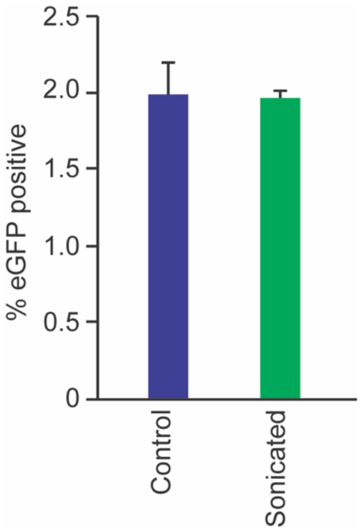

Supplemental Figure 6. Addition of energy to the system. AAV8 was loaded into alginate hydrogels in collagen-GAG scaffolds and either left to diffuse over 24 hours or left to diffuse for 24 hours before being put in a sonicator water bath for 2 hours. Media containing the releasate from the control or sonicated samples was then added HEK293t. The addition of energy did not change the AAV8 release.
